# Active core rewarming avoids bioelectrical impedance changes in postanesthetic patients

**DOI:** 10.1186/1471-2253-11-2

**Published:** 2011-02-16

**Authors:** Alma Rebeca Gutiérrez-Cruz, Bernardo Soto-Rivera, Bertha Alicia León-Chávez, Ernesto Suaste-Gómez, Daniel Martinez-Fong, Juan Antonio González-Barrios

**Affiliations:** 1 Departamento de Anestesia, Hospital Regional "Primero de Octubre", Av. IPN, No. 1669, Mexico, D. F., C.P. 07760, Mexico; 2 Área de Bioquímica y Biología Molecular, Facultad de Ciencias Qumícas, BUAP, 14 sur y Av. San Claudio, 72570, Puebla, Pue., México; 3 Departamento de Ingeniera Eléctrica, CINVESTAV, Av. IPN No. 2508, México D.F., C.P. 06760, México; 4 Departamento de Fisiología, Biofísica y Neurociencias, CINVESTAV, Av. IPN No. 2508, México D.F., C.P. 06760, México; 5 Laboratorio de Medicina Genómica, Hospital Regional "Primero de Octubre", Av. IPN, No. 1669, Mexico, D. F., C.P. 07760, Mexico

## Abstract

**Background:**

Postoperative hypothermia is a common cause of complications in patients who underwent laparoscopic cholecystectomy. Hypothermia is known to elicit electrophysiological, biochemical, and cellular alterations thus leading to changes in the active and passive membrane properties. These changes might influence the bioelectrical impedance (BI). Our aim was to determine whether the BI depends on the core temperature.

**Methods:**

We studied 60 patients (52 female and 8 male) age 40 to 80 years with an ASA I-II classification that had undergone laparoscopic cholecystectomy under balanced inhalation anesthesia. The experimental group (*n *= 30) received active core rewarming during the transanesthetic and postanesthesic periods. The control group (*n *= 30) received passive external rewarming. The BI was recorded by using a 4-contact electrode system to collect dual sets of measurements in the deltoid muscle. The body temperature, hemodynamic variables, respiratory rate, blood-gas levels, biochemical parameters, and shivering were also measured. The Mann-Whitney unpaired *t*-test was used to determine the differences in shivering between each group at each measurement period. Measurements of body temperature, hemodynamics variables, respiratory rate, and BI were analyzed using the two-way repeated-measures ANOVA.

**Results:**

The gradual decrease in the body temperature was followed by the BI increase over time. The highest BI values (95 ± 11 Ω) appeared when the lowest values of the temperature (35.5 ± 0.5°C) were reached. The active core rewarming kept the body temperature within the physiological range (over 36.5°C). This effect was accompanied by low stable values (68 ± 3 Ω) of BI. A significant decrease over time in the hemodynamic values, respiratory rate, and shivering was seen in the active core-rewarming group when compared with the controls. The temporal course of shivering was different from those of body temperatue and BI. The control patients showed a significant increase in the serum-potassium levels, which were not seen in the active-core rewarming group.

**Conclusions:**

The BI analysis changed as a function of the changes of core temperature and independently of the shivering. In addition, our results support the beneficial use of active core rewarming to prevent accidental hypothermia.

## Background

The postoperative hypothermia is a morbid condition that frequently affects patients recovering from general anesthesia [1]. Although the etiology of postoperative hypothermia is still under debate, it has long been associated with impaired metabolism, negative nitrogen balance, injury severity, hemorrhage, and multiple organ failure [2-4]. The occurrence of those dysfunctions depends on endogenous factors (endogenous hypothermia) or accidental factors (unintentional hypothermia) [4,5]. Postoperative hypothermia, in turn, may lead to severe complications such as negative catabolism and nitrogen balance, reduced resistance to infections, delayed wound healing, impaired coagulation, myocardial ischemia, and cardiac morbidities [6-8]. In particular, the cardiac complication may result in an overall higher mortality especially in patients with coronary artery disease [9]. Physiologically, the thermal input is integrated at numerous levels within the nervous system, but the hypothalamus is the dominant controller [10]. The inhibition of the thermoregulatory center in the hypothalamus by general anesthetics and sedatives is one of the causes of postoperative hypothermia, via mechanisms not completely known [11]. In contrast, the consequence of postoperative hypothermia on electrical properties especially of brain, heart, and neuromuscular activity is more characterized. Clinical evidence shows that hypothermia decreases the passive cable properties (membrane resting potential and amplitude, resistance) and impairs neuromuscular activity in such a magnitude that it can be recorded by noninvasive electrophysiological techniques [12-14].

Traditionally, bioelectrical impedance (BI) has been used as a noninvasive method to assess body-fluid compartments [15,16] and body composition [17]. The resistance, reactance, and impedance of each arm, leg, and trunk can be easily quantified by using hand and foot contact electrodes [18-20]. In the clinic, BI analysis has been successfully used to determine the cellular uncoupling triggered by an increase of intracellular Ca2+ and-or dephosphorylation of connexins during ischemia [21]. The cellular uncoupling is an important factor for the highest incidence of ventricular arrhythmia and cardiac failure [21,22]. Because the ischemia and coagulopathy are complications of prolonged hypothermia, mainly in patients with trauma or coronary artery disease [23-26], the routine monitoring of BI in hypothermic postoperative patients would be invaluable to determine the adequate treatment. Recently, the active core rewarming has demonstrated benefits of rapid rewarming of critically injured patients [27]. Therefore, we used active core rewarming in patients who had undergone laparoscopic cholecystectomy under balanced inhalation anesthesia to determine whether BI depends on the core temperature.

## Methods

The study included 60 patients (52 female and 8 male) age 40 to 80 years with an ASA I-II classification. All patients had laparoscopic cholecystectomy at the "Hospital Regional Primero de Octubre" (ISSSTE, Mexico City, Mexico) and were divided into two groups. The control group (30 patients) was treated with room-temperature parenteral solutions and a cotton blanket, which is referred to as passive external rewarming. The experimental group (30 patients) was treated with warmed parenteral solutions (38.5°C) and a cotton blanket, which is referred to as active core rewarming. This protocol was approved by the Institutional Review Board of "Hospital Regional Primero de Octubre" (ISSSTE, Mexico City, Mexico) and written, informed consent was obtained from all patients.

### Anesthesia Technique

The anesthesia procedures were done using a Dameca siesta iTS anesthesia machine (Dameca A.S., Roedovre, Denmark). All patients were treated with balanced inhalation anesthesia and complementary medication as follows; a) In the premedication phase, Midazolam (benzodiazepine); b) in the induction phase, Atropine (anticholinergic), Fentanyl (opioid), Cisatracurium (neuromuscular relaxant), and Propofol (inductors); c) in the maintenance phase, Fentanyl and Sevoflurane (volatile anesthetic agent). The dosage of all drugs was based on the body weight. The hemodynamic variables, respiratory rate, blood-gas levels, and biochemical variables were continuously measured by using a Cardiocap TM/5 monitor (Datex-Ohmeda Corp. Helsinki, Finland). During the surgical procedure, the heart rate, blood pressure, electrocardiographic trace, arterial gasometry, breathing rate, and esophageal temperature were continuously monitored. The patients treated with passive external rewarming (control group) received room-temperature parenteral solutions (1250 ± 250 mL) during the entire study. The group of patients treated with active core rewarming received their parenteral solutions (1250 ± 250 mL) warmed to 38.2°C by using a fluid warmer (Hl-90-SP-115; Smiths Medical ASD; Rockland, MA, USA) during the trans- and postsurgical period. Additional rewarming by using a cotton blanket was provided to both groups during the postanesthesia recovery. Patients treated with passive external rewarming remained in the postanesthesia care unit longer.

### Physiological Evaluation

During the trans- and postsurgical periods, homeostasis variables (blood pressure, arterial gasometry, hematic biometry, glycemia, and serum potassium levels) were evaluated three times. The first set of measurements was done before the surgical procedure; the second set, during the surgical procedure before the gallbladder was removed; and the third set, after 30 minutes of stay in the postanesthesia care unit. The body temperature (BT), respiratory rate, heart rate (HR), electrocardiography trace (EKG), mean arterial blood pressure (MABP), oxygen saturation (sO_2_), arterial carbon dioxide tension (PaCO_2_), and shivering were measured every 30 minutes for 2 hours during the stay in the postanesthesia care unit.

### Bioimpedance analysis

The BI was recorded by using a 4-contact electrode system to collect dual sets of segmental impedance measurements in the deltoid muscle. This apparatus was designed by Dr. E. Suaste-Gomez, and made by the staff of the Bioelectronics Section at CINVESTAV (Mexico City, Mexico). A constant current (0.8 mA) was applied to the lateral electrodes and the voltage drop was measured by the central electrodes. All assessments were made at 50 kHz with and 0.8 mA of constant skin current. The voltage output value was obtained by the addition of input impedance (Zo), as Zo + ΔZ (delta Z). The voltage output recorded in the two central electrodes was proportional to the input current and the impedance measured by the lateral electrode as a function of the distance between electrodes. BI measurements were made after the patient had rested 30 minutes in the postanesthesia care unit, which has a controlled temperature and relative humidity. The BI measurements were repeated every 30 minutes for 2 hours.

### Statistical analysis

All values were expressed as the mean (*s*). We determined the proper sample size (n) for BI measurements by using the equation for comparison of two means and the following values, α = 0.05; β = 0.20 with 80% of statistical power; Zα = 1.645; Zβ = 0.842; S = 30 Ω; d = 20 Ω. The Mann-Whitney unpaired *t*-test was used to determine differences in the number of patients who developed shivering between each group at each measurement period. Measurements of heart rate, mean arterial blood pressure, respiratory rate, body temperature, and BI were repeated for each group over time. Their differences were analyzed using the two-way repeated-measures ANOVA. All the statistical analysis was made using the PSPPIRE software version 0.6.0 for Linux (GNU General Public License). The null hypothesis was rejected at *P *< 0.05 level.

## Results

The age, weight, height, gender ratio, ASA classification, and duration of surgery were not statistically different between the two tested groups.

### Effect of active core rewarming on body temperature and bioelectrical impedance

**Figure 1:**
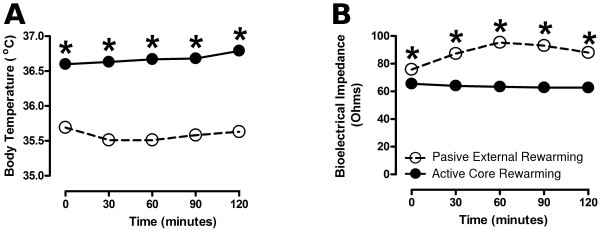
**Effect of active core rewarming on body temperature and bioelectrical impedance of postoperative patients**. Sixty adult patients underwent laparoscopic cholecystectomy under balanced inhalation anesthesia. A group of patients (*n *= 30) was treated with warmed parenteral solutions (38.5°C) and a cotton blanket. This group is referred to as Active Core Rewarming (•). The other group (*n *= 30) was treated with room-temperature parenteral solutions and a cotton blanket. This group is referred to as Passive External Rewarming (o). The values are expressed as the mean (*s*). All *P *values are < 0.001.

As expected, the body temperature in the control group remained as low as 35.5 ± 0.5°C, whereas the temperature increased gradually from 36°C to 37°C in patients treated with active-core rewarming. The temperature rise started 30 min after the onset of the treatment (Figure 1A). The patients treated with active core rewarming showed BI values of 68 ± 3 Ω, which remained stable up to the end of the testing and were significantly different to those of the patients treated with passive external rewarming (Figure 1B). In the latter group, the BI values increased from 76 ± 6 Ω to reach the highest values of 95 ± 11 Ω at 60 minutes postanesthesia and then decreased to 88 ± 9 Ω at the end of study period (Figure 1B). These values were 30% higher than those found in the group treated with active core rewarming.

### Effect of active core rewarming on physical and biochemical variables

**Figure 2:**
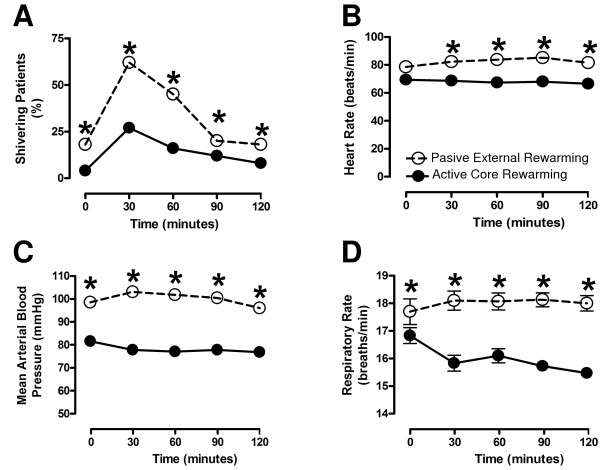
**Effect of active core rewarming on physical variables of postoperative patients**. Sixty adult patients underwent laparoscopic cholecystectomy under balanced inhalation anesthesia. A group of patients (*n *= 30) was treated with warmed parenteral solutions (38.5°C) and a cotton blanket. This group is referred to as Active Core Rewarming (•). The other group (*n *= 30) was treated with room-temperature parenteral solutions and a cotton blanket. This group is referred to as Passive External Rewarming (o). The values are expressed as the mean (*s*). All *P *values are < 0.001.

The percentage of control patients showing shivering was 25% at the beginning of the study, increased to 63% after 30 min, and then gradually decreased to 25% up to the end of the study (Figure 2A). The counts of shivering frequency were significantly decreased (50%) throughout the testing period in patients treated with active core rewarming when compared with those of the controls (Figure 2A). The heart rate of the postsurgical patients treated with active core rewarming remained stable at 67 ± 1 beats/min throughout the period of study. In contrast, the heart rate of patients who received passive external rewarming increased gradually from 77 to 85 beats/min (Figure 2B). A statistical difference was also found in the arterial blood pressure of patients treated with active core rewarming when compared with the controls. The arterial blood pressure of patients treated with active core rewarming was 20 mmHg lower than the control (Figure 2C). The mean respiratory rate of patients treated with active core rewarming was statistically lower than the control values during the period of study (Figure 2D). No significant difference between groups was found in the measurements of pH, oxygen or carbon dioxide tension, arterial-oxygen tension to the fractional inspired-oxygen ratio, arterial-oxygen saturation (*P *> 0.05), and in the biochemical variables (Table 1). The postoperative patients treated with passive external rewarming showed a significant increase in the serum-potassium levels during their stay in the anesthesia care unit (Table 1). The increase in the serum-potassium levels was not seen in the group treated with active core rewarming (Table 1).

**Table 1:** Hemodynamic and Biochemical data during pre-, trans-, and postanesthetic periods in patients after laparoscopic cholecystectomy and treated with two different rewarming techniques.

Variables	Passive External Rewarming [mean (*s)*, *n *= 30]			Active Core Rewarming [mean (*s)*, *n *= 30]		
	
	Pre-anesthesia	Trans-anesthesia	Post-anesthesia	Pre-anesthesia	Trans-anesthesia	Post-anesthesia
**HR (beats/min)**	78.8 ± 2.1	64.5 ± 1.9	78 ± 1*	79.2 ± 2.1	67.9 ± 2	71 ± 1*
**RR (breaths/min)**	15.1 ± 0.4	11.5 ± 0.17	16 ± 0	17.5 ± 0.4	11.6 ± 0.2	17 ± 0
**T°C**	36.3 ± 0.05	35.9 ± 0.06	35 ± 0	36.38 ± 0.07	36.54 ± 0.57	36 ± 0
**SABP (mmHg)**	133.9 ± 3.5	104.1 ± 3.8	126 ± 2*	124.8 ± 2.1	110.1 ± 3.1	116 ± 2*
**DABP (mmHg)**	80.7 ± 1.8	64.0 ± 1.9	77 ± 1	76.4 ± 1.8	62.3 ± 1.9	69 ± 1*
**sO_2 _(%)**	92.1 ± 0.6	98.4 ± 0.1	95 ± 0.1	92 ± 0.52	98.5 ± 0.2	96 ± 0.1
**PaCO_2 _(mmHg)**	34 ± 0.0	30.3 ± 0.6	40.2 ± 0.7	35 ± 0.4	31 ± 0.5	41.1 ± 0.4
**PaO_2 _(mmHg)**	64.6 ± 1.4	296.9 ± 1.2	76.1 ± 1.5	65.4 ± 1.8	322.9 ± 9.1	66 ± 1.4
**HCO_3 _(mmol/L)**	20.6 ± 0.2	17.78 ± 0.5	20.5 ± 0.3	21.6 ± 0.2	19.7 ± 0.3	21.13 ± 0.21
**Hgb (mg/dL)**	13.4 ± 0.3	12.8 ± 0.3	12.6 ± 0.3	13.5 ± 0.3	13.1 ± 0.3	12.9 ± 0.3
**Hct (%)**	42.9 ± 1	41 ± 1	39.9 ± 0.1	42.6 ± 0.3	40.4 ± 0.9	40 ± 1
**Leukocytes (10^3^/cm³)**	7.5 ± 2.8	7.3 ± 2.8	7.3 ± 2.8	7.9 ± 2.8	7.7 ± 3.1	7.8 ± 2.7
**Platelets (mm³)**	283567 ± 13030	274200 ± 12495	271200 ± 12818	326500 ± 9088	326467 ± 9007	326333 ± 9028
**pH**	7.41 ± 0.01	7.35 ± 0.01	7.30 ± 0.01	7.38 ± 0.01	7.38 ± 0.01	7 ± 0.01
**K (mEq/L)**	3.57 ± 0.04	3.96 ± 0.03*	4.08 ± 0.03*	3.58 ± 0.03	3.51 ± 0.03*	3.45 ± 0.04*
**Glucose (mg/dL)**	95.87 ± 4.8	109.4 ± 5.4*	116.5 ± 5.2	93.7 ± 3.5	88.7 ± 4.2*	79.8 ± 2.7*

Data are expressed as mean (*s*) *Significant differences between active-core rewarming and passive-external rewarming (*P *< 0.05). HR, heart rate; HCO_3_, bicarbonate; SABP, systolic arterial blood pressure; DABP, diastolic blood pressure; sO_2_, oxygen saturation; PaCO_2_, arterial carbon dioxide tension; PaO_2_, arterial oxygen tension; RR; respiratory rate; Hgb, hemoglobin; Hct, hematocrit; K, potassium; T°C, body temperature in Celsius.

## Discussion

Our results show for the first time that the BI is directly affected by the changes of core temperature in postoperative patients subjected to laparoscopic cholecystectomy. We showed that the BI values are inversely associated with the changes in the body temperature, but not with the temporal course of shivering. The gradual decrease in the body temperature led to the increase in the BI values. The highest BI value (95 ± 11 Ω) appears at 60 min when the lowest temperature (35.5 ± 0.5°C) is reached. Furthermore, the active core rewarming maintained stable physiological values of body temperature (over 36.5°C), which were associated with stable low values of the BI (68 ± 3 Ω) over time. An increasing number of works show that the BI analysis is a reliable method to determine the fluid and body composition, and the cellular uncoupling in pathological conditions [16,19,21]. On this basis, we suggest that the increase in BI in postoperative hypothermic patients reflects the development of physiological, biochemical and cellular alterations. The precise molecular composition in fluid and body and the physiological mechanism underpinning BI changes in hypothermic patients remain to be determined.

Hypothermia is usually caused by a prolonged time of CO_2 _insufflation during the laparoscopic surgery [28]. In addition, the anesthetics, sedatives, and muscle relaxants are other factors able to potentiate the effect of CO_2 _insufflation on hypothermia during surgery [11]. These drugs are able to depress the hypothalamic thermoregulation and generate shivering as a compensatory mechanism [10]. In our study, the long surgical time (82 ± 5 min) as compared with others [29] can account for the sustained hypothermia, the increased hemodynamic values, and the shivering observed in patients who received passive external rewarming. The increased values of hemodynamic variables in the hypothermic patients of this study could result from the adrenergic response to hypothermia, which has been supported by others [30,31]. It has long been known that the adrenergic response caused by the increased release of norepinephrine in response to cold causes vasoconstriction and secondary hypertension [32,33]. In our study, the postoperative patients treated with passive external rewarming also showed a mild but significant increase in the serum-potassium levels during the trans- and postanesthetic period. This alteration is possibly caused by an overstimulation of the neuromuscular junction and ectopic contractions in shivering patients. Similar results have been found in electroconvulsive therapy, in which there is a fast increase of plasmatic potassium levels [34].

In agreement with previous results [35], the active core rewarming used in this study caused stable values of body temperature near the physiological range (over 36.5°C) along with a significant decrease in the hemodynamic values and shivering. These results suggest that the control of core temperature is able to prevent the adrenergic response and maintain the passive electrical properties within the physiological range. On this basis, we suggest that the BI changes depend on the core temperature changes. Because of this property, the BI may be a useful tool to monitor the recovery from hypothermia in postoperative patients, especially for those with cardiac antecedents [22]. In addition to routine monitoring, the BI data might lead to more timely interventions, resulting in clinical improvement and a shorter stay in the anesthesia care unit.

## Conclusions

The BI changed as a function of the core temperature and independently of the involuntary muscle activity. The physiological and molecular mechanisms underpinning the BI changes in hypothermic postoperative patients require further studies. In addition, our results support the beneficial use of active core rewarming to prevent accidental hypothermia and changes in BI.

## Competing interests

The authors have no financial and personal relationships with other people, or organizations that could inappropriately influence their work, all within 3 years of beginning the work submitted.

## Authors' contributions

ARG made the anesthesia procedure, and participated in the collection and analysis of the data. BSR participated in the data collection and analysis. BALCH participated in the data analysis. ESG designed the 4-contact electrode system recorder. DMF was the scientific advisor and prepared the final version of this manuscript. JAGB participated in the design of the study, in the data analysis, conceived the paper, and prepared the figures, table, and the final version of this manuscript. All authors read and approved the final version of the manuscript.

## Consent

Each patient for publication freely gave a written informed consent. A copy of the written consent is available to the Editor-in-Chief of this Journal.

## Pre-publication history

The pre-publication history for this paper can be accessed here:

http://www.biomedcentral.com/1471-2253/11/2/prepub
